# The effect of radiographic vertebral body and intervertebral disc wedging on curve progression in idiopathic scoliosis

**DOI:** 10.1186/1748-7161-8-S1-O38

**Published:** 2013-06-03

**Authors:** R Volz, LA Dolan, F Masrouha, SL Weinstein

**Affiliations:** 1 University of Iowa, IA, USA

## Background

Wedging of the apical vertebral body and the intervertebral disc are well-known characteristics of adolescent idiopathic scoliosis (AIS). Development of AIS may be secondary to vertebral wedging caused by primary abnormalities of the vertebral growth plate. Several studies have examined the relationship of vertebral body and disc wedging with scoliosis curve progression.

## Aim

To estimate the reproducibility of apical vertebral and disc wedging, the correlation between wedging and Cobb angle, and the role of vertebral wedging in curve progression after skeletal maturity.

## Methods

Baseline and 30-year follow-up x-rays from 56 AIS patients were evaluated. Wedging measurements included apical vertebral body height ratios (VBHR), apical vertebral body angles (VBA) and apical intervertebral disc wedging angles (IVDA). Intra- and inter-rater reliability was estimated using 2 readers and a subset of 20 curves. Multivariate regression estimated the contribution of wedging to prediction of curve progression.

## Results

Intra-rater tolerance limits were: VBHR ±12%; VBA ±7°; IVDA ±7°. Inter-rater tolerance limits were significantly larger: VBHR ±23%; VBA ±11°; IVDA ±11°. Cobb angles were moderately correlated with wedging at baseline (VBHR -0.51; VBA 0.46; IVDA 0.40) and with the VBHR at follow-up (-0.65). Average curve progression was 18° (range -11° - 126°). The average change in VBHR over time was ~5%. Adding VBHR to a regression equation including baseline Cobb angle, age, Risser and years of follow-up improved prediction of the future Cobb angle (increase in adjusted R2 from 0.65 to 0.71).

## Conclusions

This study provides tolerance limits to judge if a true change in apical vertebral or apical disc wedging has occurred. These limits should be considered when evaluating bracing or other procedures which potentially unload the spine and affect wedging. Wedging measured using vertebral height ratios was much more reliable than when measured using angles. Despite the average curve progression of 18°, there was no appreciable change in vertebral wedging over time. Clinicians and researchers should consider including vertebral height ratios when estimating long-term curve progression in AIS patients.
